# Knockout of *Arabidopsis thaliana* *VEP1*, Encoding a PRISE (Progesterone 5β-Reductase/Iridoid Synthase-Like Enzyme), Leads to Metabolic Changes in Response to Exogenous Methyl Vinyl Ketone (MVK)

**DOI:** 10.3390/metabo12010011

**Published:** 2021-12-23

**Authors:** Jan Klein, Mona Ernst, Alexander Christmann, Marina Tropper, Tim Leykauf, Wolfgang Kreis, Jennifer Munkert

**Affiliations:** 1 Department of Plant Physiology, Matthias-Schleiden-Institute for Genetics, Bioinformatics and Molecular Botany, University of Jena, 07743 Jena, Germany; jan.klein@uni-jena.de; 2 Department of Biology, University of Erlangen-Nuremberg, 91058 Erlangen, Germany; mona.ernst@gmx.de (M.E.); marina.tropper@fau.de (M.T.); tim.leykauf@fau.de (T.L.); wolfgang.kreis@fau.de (W.K.); 3 Chair of Botany, TUM School of Life Sciences Weihenstephan, Technical University of Munich, 85354 Freising, Germany; alexander.christmann@tum.de

**Keywords:** *Arabidopsis thaliana*, steroid reductase, reactive electrophile species, specialized metabolism

## Abstract

Small or specialized natural products (SNAPs) produced by plants vary greatly in structure and function, leading to selective advantages during evolution. With a limited number of genes available, a high promiscuity of the enzymes involved allows the generation of a broad range of SNAPs in complex metabolic networks. Comparative metabolic studies may help to understand why—or why not—certain SNAPs are produced in plants. Here, we used the wound-induced, vein patterning regulating *VEP1* (*At*StR1, At4g24220) and its paralogue gene on locus At5g58750 (*At*StR2) from *Arabidopsis* to study this issue. The enzymes encoded by *VEP1*-like genes were clustered under the term PRISEs (progesterone 5β-reductase/iridoid synthase-like enzymes) as it was previously demonstrated that they are involved in cardenolide and/or iridoid biosynthesis in other plants. In order to further understand the general role of PRISEs and to detect additional more “accidental” roles we herein characterized *A. thaliana* steroid reductase 1 (*At*StR1) and compared it to *A. thaliana* steroid reductase 2 (*At*StR2). We used *A. thaliana* Col-0 wildtype plants as well as *VEP1* knockout mutants and *VEP1* knockout mutants overexpressing either *At*StR1 or *At*StR2 to investigate the effects on vein patterning and on the stress response after treatment with methyl vinyl ketone (MVK). Our results added evidence to the assumption that *At*StR1 and *At*StR2, as well as PRISEs in general, play specific roles in stress and defense situations and may be responsible for sudden metabolic shifts.

## 1. Introduction

Specialized metabolism, sometimes still termed “secondary metabolism”, has been studied extensively in plants and microorganisms. In the twentieth century, the vast chemical diversity of the natural products formed by plants attracted organic chemists even more than plant biologists. A lot of the complex natural products appearing in certain taxa during different developmental stages were labelled as “secondary” metabolites because a fundamental role in cell metabolism and growth was not seen. Today, several small (or specialized) natural plant products (SNAPs), such as jasmonates, brassinosteroids, and strigolactones have lost their stigma of being secondary metabolites. They are now regarded as important regulatory elements of plant development and growth. In some instances, it is difficult to determine which metabolite is “secondary” and which is “primary”, particularly when they share a common biosynthetic route and the same structural scaffold. Examples are psilocybin (a toxic alkaloid) and indole-3-acetic acid (a phytohormone), both derived from tryptophan, or β-sitosterol (a regulator of membrane fluidity in plants) and digitonin (a toxic saponin) both formed via the mevalonic acid pathway. A “secondary” pathway leading to just a single specific natural product therefore does not seem very probable. SNAP formation depends on the availability of suitable substrates and enzymes able to modify them. A relaxed substrate specificity of many enzymes enhances a plant’s options to develop complex metabolic grids, because a limited number of genes is available in a plant’s genome to encode the respective enzymes. This is also the basis for substrate–enzyme coevolution, which has been demonstrated as the fundamental principle leading to the formation of SNAPs [1,2]. Ecological and environmental factors might lock in a certain SNAP profile for a time and allow the taking of metabolic snapshots using modern tools of comparative metabolism, such as metabolomics. Comparative metabolism studies help with understanding and predicting the metabolic fate of pharmaceuticals and other xenobiotics in animals but may also do so in plants [3]. For example, they are advised to be conducted in pesticide risk assessment [4]. Other than studies related to the metabolism of xenobiotics, comparison of genes, enzymes and metabolites of plants that either do or do not produce a certain group of SNAPs provides a new aspect for comparative metabolism. This approach may help to understand the more general roles of enzymes assumed to be mainly responsible for the production and accumulation of certain natural compounds. 

The *Arabidopsis thaliana* gene *VEP1* (At4g24220) [5], also described as *AWI 31* [6], is ideal to use in comparative metabolism studies. Wounding or environmental stressors enhanced *VEP1* expression in *A. thaliana* [6]. Gene knockouts developed a phenotype with altered vein patterning [5]. Based on *in silico* analyses only one other locus (At5g58750) was identified within the genome of *A. thaliana* encoding a related protein [7]. The enzymes encoded by At4g24220 and At5g58750 were termed *At*StR1 (*A. thaliana* steroid reductase 1) and *At*StR2 (*A. thaliana* steroid reductase 2), because they reduced the double bond of Δ4,5-steroids and other α,β-unsaturated ketones [8,9]. All enzymes encoded by *VEP1*-like genes were grouped under the name PRISEs (progesterone 5β-reductase/ iridoid synthase-like enzymes) [10]. In the plant progesterone 5β-reductase family, iridoid synthase activity was commonly detected [7].

PRISEs came into focus in specialized plant metabolism because they act as progesterone 5β-reductases in 5β-cardenolide biosynthesis [11,12,13,14] and iridoid synthases in iridoid biosynthesis [7,15]. *RNAi*-mediated gene knockdown of PRISEs led to reduced cardenolide contents in *Digitalis lanata* [14]. The direct participation of PRISEs in iridoid biosynthesis was demonstrated in virus-induced gene silencing (VIGS) experiments using *Catharanthus roseus* [15]. It was only recently proposed that PRISEs could provide a catalytic reservoir for specialized metabolism across all land plants [16].

*VEP1*-like genes appeared early in plant evolution, and it was assumed that a gene transfer from α-proteobacteria ultimately helped early plants to adapt to life on land [17]. A recent database search yielded hits for *VEP1*-like genes in 198 species of seed plants, most of them containing neither cardenolides nor iridoids, including *A. thaliana* [9]. However, the *VEP1*-encoded *At*StR1 converted progesterone, a precursor of cardenolides, 20 times more efficiently than the corresponding *Digitalis lanata* enzyme [8] and it converted other small molecules even more efficiently than progesterone [18,19], and furthermore iridoid precursors were also accepted as substrates [7,9].

**Figure 1:**
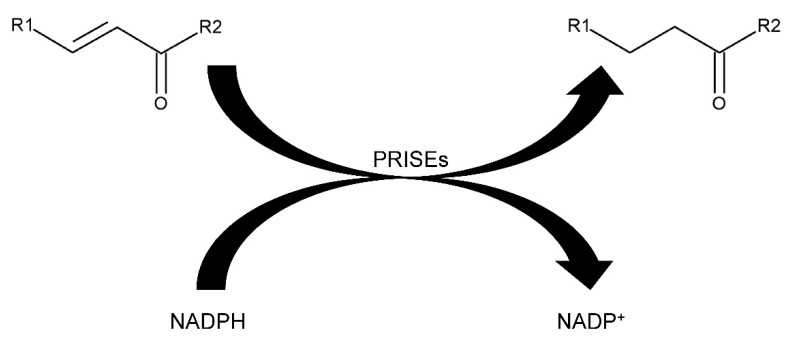
Detoxification of reactive electrophile species (RES) by PRISEs. R_1_ = R_2_ = CH_3_: methyl vinyl ketone (3-buten-2-one) is converted to 2-butanone.

PRISEs can detoxify reactive electrophile species (RES), such as methyl vinyl ketone (MVK) (Figure 1), a product of trienoic fatty acid peroxidation [20], very efficiently [7,9]. *VEP1* gene products may have enhanced the pathogenicity of α-proteobacteria by interrupting defense-related signal cascades involving RES. This may have assisted the plants conquering land to survive in an oxygen-containing atmosphere that promoted lipid peroxidation without a pathogen attack [17]. 

MVK is a powerful inducer of defense-related gene expression [21] and PRISEs may therefore modify stress responses through metabolizing MVK and related compounds. Consequently, a more general physiological role for PRISEs has been proposed in stress responses and detoxification processes [9,14].

In order to investigate the general role of PRISEs and to elucidate their “accidental” roles we characterized *At*StR1 and compared it to *At*StR2. We used *A. thaliana* Col-0 wildtype (wt) plants as well as *At*StR1 knockout mutants (*At*StR1-) and knockout mutants overexpressing either *At*StR1 (*35S:At*StR1/*At*StR1-) or *At*StR2 (*35S:At*StR2/*At*StR1-) to investigate the effects of MVK treatment.

## 2. Results and Discussion

### 2.1. Characterization of AtStR2

**Figure 2:**
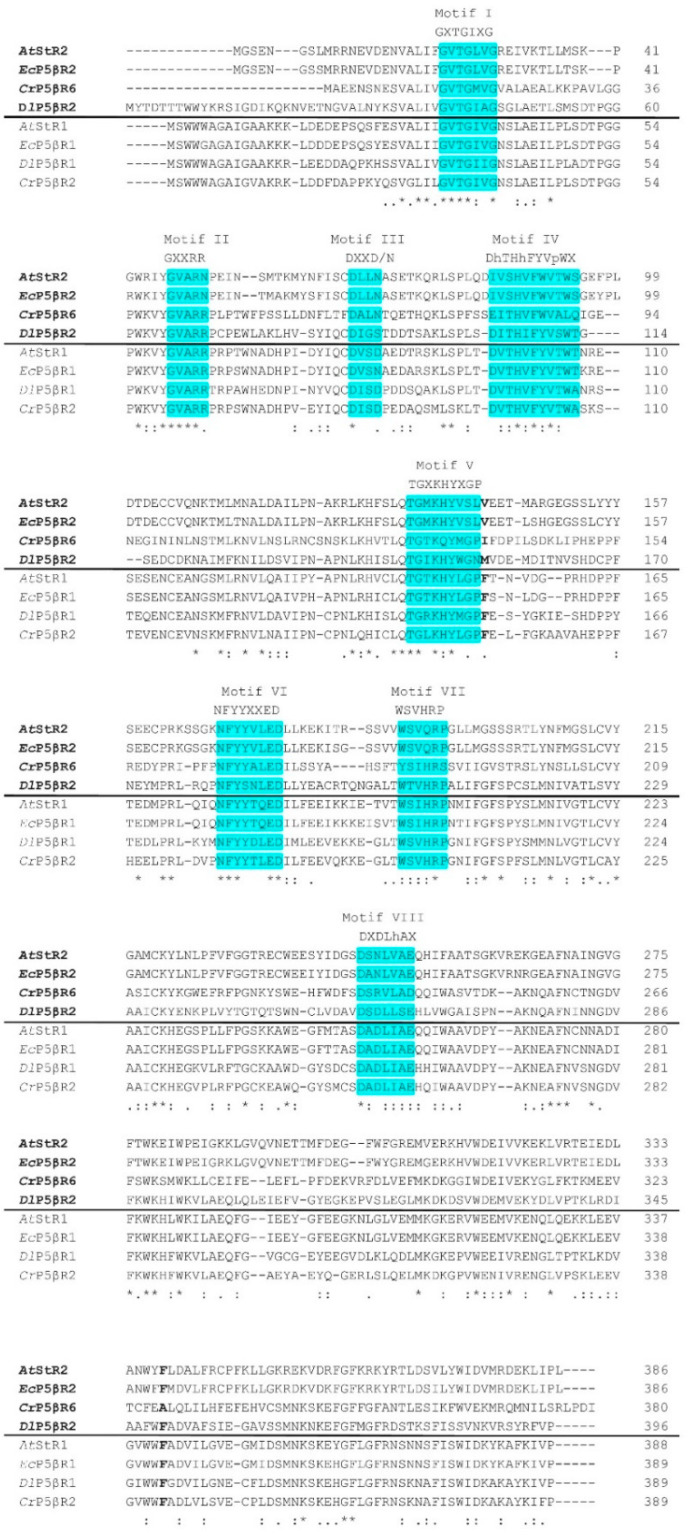
Amino acid alignment, performed by ClustalW, of several PRISEs. Bold: PRISEs of cluster II, namely, *At*StR2 (NP_200683.1), *D. lanata Dl*P5βR2 (ADL28122.1), *E. crepidifolium Ec*P5βR2 (AGT29343.1) and *C. roseus Cr*P5βR6 (AIW09148.1). Light: PRISEs of cluster I, namely, *A. thaliana At*StR1 (NP_194153.1), *E. crepidifolium Ec*P5βR1 (ADG56544.1), *D. lanata Dl*P5βR1 (Q6PQJ9.1) and *C. roseus Cr*P5βR2 (AIW09144.1). The motifs described by [23,24] are shown in turquoise shading. The residues of the phenylalanine clamp defined by [10] are depicted in bold. Asterisk, fully conserved; colon, strongly conserved; period, weakly conserved.

*At*StR2 is encoded by locus At5g58750. An appropriate cDNA was deduced and cloned into a pDEST17 expression vector and then functionally expressed in *E. coli*. The recombinant enzyme was termed r*At*StR2. It was purified (Figure S1) and then used for enzyme characterization. We demonstrated that r*At*StR2 converted progesterone enantioselectively into 5β-pregnane-3,20-dione (Figure S2). Progesterone was converted equally well by r*At*StR2 and r*At*StR1, but *At*StR1 converted MVK much better than r*At*StR2 (Table 1). *At*StR1 and *At*StR2 were aligned together with other members of the PRISE family using ClustalW [22] and all PRISE motifs described by [23,24] could be identified (Figure 2). 

Motifs I to III are typically present in all NADP^+^-dependent SDRs, the protein family to which the PRISEs belong. NADPH_2_ but not NADH_2_ was accepted as a cosubstrate by *At*StR2 (Table 1). In all PRISEs investigated so far, NADPH_2_ was the preferred cosubstrate. Cosubstrate specificity in *At*St5βR muteins was previously changed with limited success modifying the typical NADPH_2_-binding motif GVARR [25]. 

*At*StR2 shares 92% amino acid identity with a PRISE from *Erysimum crepidifolium* (AGT29343.1). This high interspecific similarity and the much lower intraspecific similarity (less than 40% identity between *At*StR1 and *At*StR2, as well as *Ec*P5βR1 and *Ec*P5βR2, respectively) led to the assumption that at least two clusters (cluster I and cluster II) of PRISEs needed to be differentiated [7]. Only two *PRISE* genes were identified in the genome of *A. thaliana*, whereas up to seven *VEP1*-like genes were reported in other plant genomes [9]. Examples of cluster I and cluster II PRISEs are shown in Table 1. When the substrate spectra of *At*StR1 and other cluster I PRISEs were studied, it was demonstrated that these enzymes converted small RES more efficiently [7,9,19]. Methyl vinyl ketone (MVK) belongs to this group of compounds. When calculating the progesterone/MVK quotient (Q_P/M_) of the catalytic efficiencies shown in Table 1, it became obvious that PRISEs of cluster I usually prefer MVK over progesterone as a substrate, indicated by a smaller Q_P/M_ (for example, Q_P/M_ *At*StR1 = 0.29 but Q_P/M_ *At*StR2 = 14.20 and Q_P/M_ *Ec*P5βR1 = 0.01 but Q_P/M_ *Ec*P5βR2 = 2.25).

Petersen et al. [10] generated recombinant *At*StR1 muteins that had almost or completely lost their capacity to convert MVK and small cyclic 1,4-enones, such as cyclocexen-1-one. These muteins preferred larger substrates such as progesterone, a precursor of 5β-cardenolides (Table 1) and 8-oxogeranial, a precursor of iridoids instead [10]. 

This was achieved by the removal of the phenylalanines forming a clamp structure at the rim of the binding pocket [9]. With the disruption of this feature, small, rather hydrophilic 1,4-enones may not be “trapped” in the binding pocket long enough to allow for the conversion [9,10]. Since the phenylalanine clamp is highly conserved and found in most of the class I PRISEs described so far, it seems as if these enzymes were not optimized for larger molecules such as progesterone [9].

**Figure 3:**
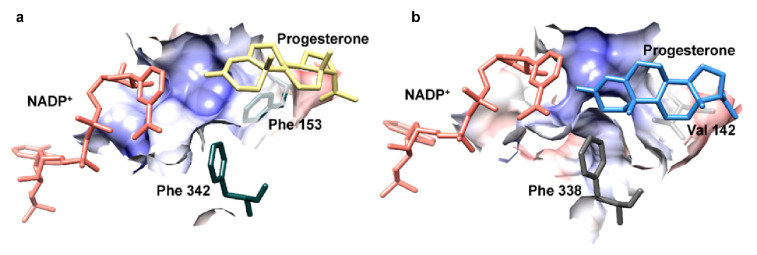
Docking of progesterone into the active site of *At*StR1 (**a**) and homology-modelled *At*StR2 (**b**). The coulombic surface is displayed for important residues in the active site according to [32]. NADP^+^ (red), progesterone (yellow for *At*StR1, blue for *At*StR2) and the phenylalanine clamp residues (green for *At*StR1, grey for *At*StR2) are shown as stick models.

To analyze this in more detail, we here modeled *At*StR2 on the crystal structure of *At*StR1 (PDB entry 6el3.1.A) cocrystallized with NADP^+^ [9], which is available in the RSCB protein data bank [28]. The model was created using Swissmodel [29] and progesterone was docked into the active site (Figure 3) with AutoDock Vina [30]. The results were visualized in USCF Chimera [31] and demonstrated a different fit of progesterone in the active site of *At*StR1 and *At*StR2, respectively.

In *At*StR2, as well as in other cluster II PRISEs, the phenylalanine adjacent to motif 5 was substituted for other amino acids, hence a phenylalanine clamp could not be formed. As a consequence, the active site could accommodate larger molecules such as progesterone (Figure 3).

The loss of activity regarding small molecules, which cannot be trapped in the active site has already been described [10]. Their interpretation of the important role of a phenylalanine clamp at the rim of the binding pocket is consistent with the observation that the cluster II PRISEs we analyzed here (Figure 2) lack at least one of the two phenylalanines. This explains their lower catalytic efficiency for MVK. With this in mind, the r*At*StR1 mutants r*At*St5βR1_F153A and r*At*St5βR1_F153A_F342A [10] could be regarded as cluster I PRISEs converted to cluster II PRISEs. The substrate preferences of r*At*StR1, and its mutants have already been characterized quite well [10,18,19] whereas more data are still required for r*At*StR2.

Paralogues obtained after gene duplication may have been formed in metabolite–enzyme coevolution [2] towards the preference of larger molecules and the discrimination of small RES released in stress reactions. Other substrate-promiscuous enzymes such as BAHD-type malonyltransferases may also have morphed from enzymes capable of detoxifying xenobiotics to enzymes capable of malonylating 21-hydroxypregnanes, yielding precursors of butenolide ring formation in the cardenolide biosynthesis [33]. In a similar way, PRISEs may have evolved helping to establish cardenolide formation in *E. crepidifolium*, a close relative of *A. thaliana*.

### 2.2. Expression of AtStR1 and AtStR2

Here, we established and analyzed three systems: (i) *Ath* Col-0 wt (wild type plants), (ii) homozygous SALK 097798 knockout plants (*At*StR1-) and (iii) SALK 097798 expressing either *At*StR1 (*35S:At*StR1/*At*StR1-) or *At*StR2 (*35S:At*StR2/*At*StR1-) under the control of the CaMV 35S promotor. Homozygous knockout plants were identified using primers designed by the “SALK T-DNA verification primer design”.

**Figure 4:**
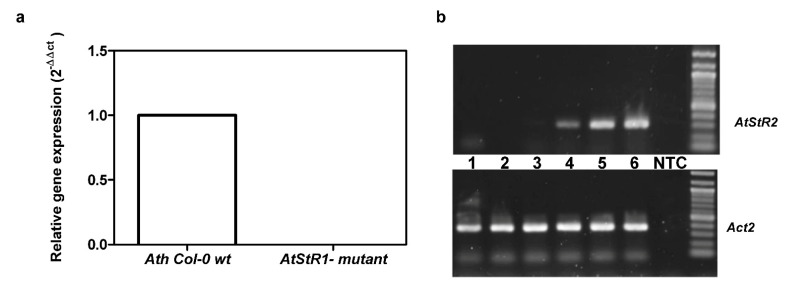
Expression of *At*StR1 in *Ath* Col-0 wt and *At*StR1- mutant (**a**) and expression of *At*StR2 in adult plant leaves and in seedlings (**b**). (**a**) *At*StR1 is expressed in *Ath* Col-0 wt but not in *At*StR1- mutant (Salk 097798). (**b**) *At*StR2 is strongly expressed in seedlings (4/5/6) but poorly in leaves of adult plants (1/2/3) as confirmed by normal PCR. *Act2* and *EF1α* were used as reference genes in a quantitative real-time PCR experiment (*n* = 3) and *Act2* was used as control gene in the semiquantitative PCR. NTC = non template control.

*At*StR1 was expressed in *Ath* Col-0 wt but not in *At*StR1- mutant plants, verifying the stable knockout of *At*StR1 in the homozygous plants used here (Figure 4). *At*StR2 was strongly expressed in seedlings (Figure 4b) and cold-treated adult leaves (Figure S3) but poorly in adult leaves of non-stressed *A. thaliana* plants (Arabidosis eFP browser 2.0; Figure 4a and Figure S3). Since *At*StR2 is not expressed in leaves (in normal conditions), *At*StR2 activity did not interfere with the determination of *At*StR1 activities in the enzyme assays of leaf extracts (Figure S4). Other enzymes possibly converting progesterone to 5β-pregnane-3,20-dione seemed to be inactive (Figure S4).

**Figure 5:**
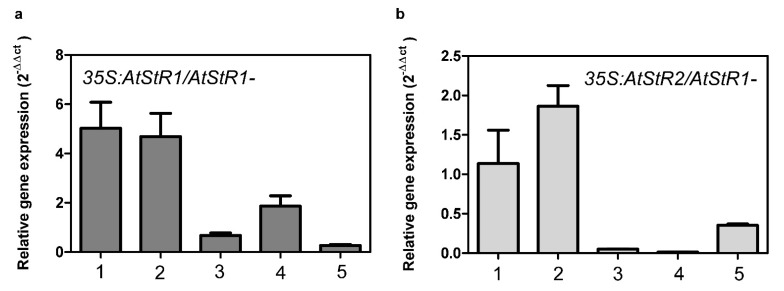
Expression of *At*StR1 gene in transgenic *35S:At*StR1/*At*StR1- (**a**) and expression of *At*StR2 gene in *35S:At*StR2/*At*StR1- mutant lines (**b**). mRNA values were measured by quantitative real-time PCR, quantified by 2^−ΔΔct^method using *Act2* and *EF1α* as reference genes (*n* = 3). Numbers 1–5 indicate individual lines *35S:At*StR1/*At*StR1-1–5 and *35S:At*StR2/*At*StR1-1–5.

PRISE expression of five individual transgenic lines each (*35S:At*StR1/*At*StR1- and *35S:At*StR2/*At*StR1-) were analyzed. The two lines of each approach showing the highest expression rates were chosen for further experiments. These lines were termed *35S:At*StR1/*At*StR1-1, *35S:At*StR1/*At*StR1-2, *35S:At*StR2/*At*StR1-1 and *35S:At*StR2/*At*StR1-2 (Figure 5).

Insertion lines in which *At*StR2 on locus At5g58750 was inactivated were not available to us. Attempts to generate plants lacking the *At*StR2-encoding gene using either RNAi-mediated gene knockdown or CRISPR/Cas9-mediated gene knockout have been unsuccessful. It is possible that *At*StR2 might be essential for plant development and a lack of it stops seed germination or seedling development.

Progesterone 5β-reductase activity was recovered in the *35S:At*StR1/*At*StR1- as well as in *35S:At*StR2/*At*StR1- lines, indicating that the respective enzymes were produced in an active form (Figure S4).

We investigated the effects of MVK treatment on *Ath* Col-0 wt and mutant plants, because MVK is a powerful inducer of defense-related gene expression [21] and PRISEs could be involved in the stress response simply by metabolizing MVK itself. We analyzed surrogate parameters for the stress response, such as H_2_O_2_ levels and *glutathione reductase* (*GR1*) expression. Glutathione reductase (GR1) is an enzyme influencing the redox state of a cell by releasing reduced glutathione (GSH) from oxidized glutathione (GSSG) [34]. Redox states can therefore be evaluated by measuring *GR1* expression [14].

**Figure 6:**
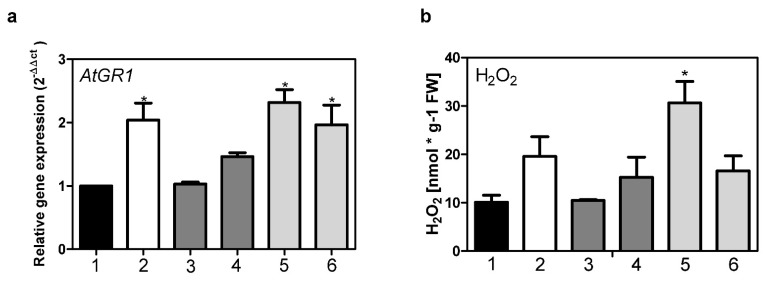
Expression of *glutathione reductase (GR1)* in leaves of *A. thaliana* Col-0 wt and mutant lines (**a**) and quantification of H_2_O_2_ levels (**b**) in *A. thaliana* Col-0 wt and mutant lines. (1) *Ath* Col-0 wt, (2) *At*StR1-, (3) *35S:At*StR1/*At*StR1-1, (4) *35S:At*StR1/*At*StR1-2, (5) *35S:At*StR2/*At*StR1-1, (6) *35S:At*StR2/*At*StR1-2. Mean ± STD (*n* = 3), normalized on *Act2* and *EF1α* (* = *p* < 0.05).

A*tStR2* was not induced under MVK stress and r*At*StR2 only poorly accepted MVK (Table 1). Moreover, the redox state as estimated by *GR1* expression and H_2_O_2_ production was not altered in *At*StR1- mutants transformed with *35S:At*StR2 (Figure 6). The *A. thaliana* glutathione reductase gene *GR1* is expressed twice as high in *At*StR1- mutants than in *Ath* Col-0 wt. H_2_O_2_ levels were also higher in *At*StR1- mutants than in *Ath* Col-0 wt (Figure 6). This indicates reduced GSH availability. The effect could be reduced to wild type levels after curing the *At*StR1- mutant with *35S:At*StR1 but not with *35S:At*StR2. A disturbed redox state, indicated by a changed expression of a cytosolic glutathione reductase and increased H_2_O_2_ levels, was also seen in *D. lanata* RNAi shoot culture lines in which *VEP1*-like genes were knocked down. In these mutants, four times higher GSH levels compared to wild type controls have been demonstrated [14].

### 2.3. Roles of AtStR1 and AtStR2 in Plant Reactive Electrophilic Species (RES) Stress

Cardenolide and/or iridoid pathways are not known to be present in *A. thaliana*. Iridoids are not known to occur in the Brassicaceae and only few genera, including *Erysimum*, accumulate cardenolides. *At*StRs cannot be involved in pathways that are not existent in *A. thaliana*. Consequently, though capable of reducing the double bond of progesterone enantioselectively, here PRISEs simply cannot be connected to 5β-cardenolide formation. Enzymes as well as pregnane precursors may, however, be part of a “silent metabolism” in which putative intermediates and enzymes are present that have not yet been detected or are not associated with known metabolic pathways [1,35]. Studies in comparative metabolism (including metabolomics techniques) may help to better understand and evaluate these “silent” resources.

Small reactive carbonyl species, also known as reactive electrophilic species (RES) formed during stress reactions, have already been identified as putative substrates for PRISEs. Examples are small 1,4-enones such as methyl vinyl ketone (MVK) or 1-cyclohexen-2-one which are converted by *At*StR1 very efficiently [7,9,10,19]. MVK is a product of trienoic fatty acid peroxidation [20]. MVK accumulates in various stress situations [10,36,37,38] and is partly responsible for heat stress symptoms in cyclamen [20]. It is known that MVK causes tissue damage in *A. thaliana* [38] and *Cyclamen persicum* [20] leaves. The enzymatic reduction of MVK yields methyl ethyl ketone (MEK) which is harmless to plants [38]. There are other enzymes and mechanisms known to detoxify or inactivate RES. For example, RES can be detoxified by spontaneous reaction or enzymatic conjugation with reduced glutathione (GSH) [39]. RES, specifically 1,4-enones can also be reduced by aldehyde oxidoreductases (AOR) and 2-alkenal reductases (AER). We here considered these options as alternative routes for MVK detoxification and analyzed the expression pattern of the respective *A. thaliana* genes.

*VEP1*, *AER* and *AOR* are differentially expressed in *A. thaliana*. In the absence of stress, *AER* is expressed only weakly, whereas *AOR* is strongly expressed in young and *VEP1* in mature leaves (Arabidopsis eFP browser 2.0). The respective enzymes differ in their substrate preferences, especially the size of potential substrates. For example, an AOR of *Cucumis sativa* reduced 4-hexen-3-one about 100 times more efficiently than MVK [27]. On the other hand, the *At*AOR characterized in the same paper was equally active towards these two substrates. The authors discussed that AOR together with other enzymes might cooperatively detoxify reactive carbonyls. We here provide evidence that *VEP1*-encoded PRISEs may also be important players in this scenario.

**Figure 7:**
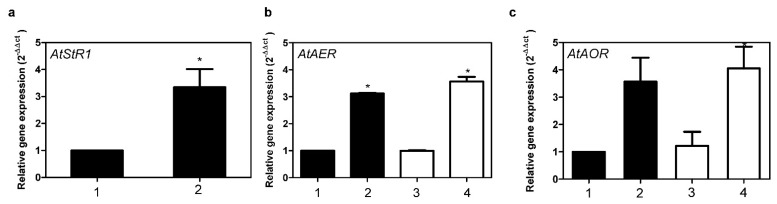
Expression of *At*StR1 (**a**), *AtAER* (**b**) and *AtAOR* (**c**) in *A. thaliana* leaves. (**1**) *Ath* Col-0 wt, (**2**) *Ath* Col-0 wt + MVK, (**3**) *AtStR-* mutant, (**4**) *AtStR-* mutant + MVK. Mean ± STD (*n* = 3), normalized on *Act2* and *EF1α* (* = *p* < 0.05).

*AtAER* (At5g16970) encodes a cytosolic alkenal reductase in *A. thaliana*. As far as the kinetic constants are concerned *At*StRs and *At*AER have similar *K*_m_ values but differ in their catalytic efficiency, *At*AER being about 200 times more efficient than *At*StR1. Expression of *AtAER* and *AtAOR* was equally high in *Ath* Col-0 wt and in *At*StR1- mutants and could be amplified by MVK, in this way paralleling enhanced *At*StR1 expression (Figure 7). Since *AtAER* is only weakly expressed in leaves and is enhanced moderately only under MVK stress, the strong expression of *At*StR1 may lead to an amount of enzyme that can efficiently compete with *At*AER for MVK detoxification. *AtAOR (*At1g23740), in contrast to *AtAER*, is strongly and constitutively expressed in the chloroplast and is assumed to be involved in the general elimination of reactive carbonyls [27]. All in all, detoxification of reactive carbonyls seems to be a more complex situation with correlating enzymes working in a group and providing a detoxifying network system. However, this issue requires further investigation. A 3 h exposure to MVK resulted in a fourfold increase of *At*StR1 expression in *Ath* Col-0 wt (Figure 7). Expression stayed high even after 24 h of treatment (data not shown). On the other hand, *At*StR2 was neither expressed in the control plants nor was its expression induced after MVK treatment, even after prolonged exposure up to 24 h.

**Figure 8:**
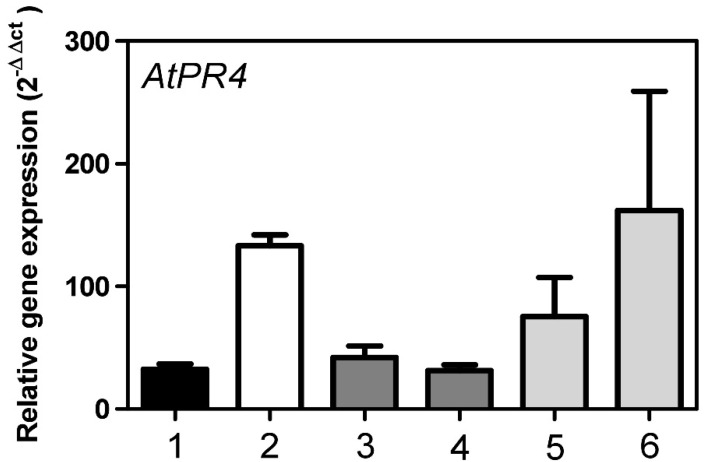
Expression of *AtPR4* in *A. thaliana* leaves under MVK treatment in relation to *AtPR4* expression in untreated *Ath* Col-0 wt. (1) *Ath* Col-0 wt, (2) *At*StR1-, (3) 35S:*At*StR1/*At*StR1-1, (4) 35S:*At*StR1/*At*StR1-2, (5) 35S:*At*StR2/*At*StR1-1, (6) 35S:*At*StR2/*At*StR1-2. Mean ± STD (n = 3), normalized on *Act2* and *EF1α* (* = *p* < 0.05).

The *AtPR4* gene (also termed *hel* gene) has successfully been used as a surrogate to estimate MVK effects [21,40,41]. *AtPR4* encodes a PR (pathogen-related) protein. PR proteins are induced in response to pathogen attacks and other stresses. When treated with MVK, *AtPR4* was expressed around five times higher in *At*StR1- mutants than in *Ath* Col-0 wt. This difference was taken as an indication for impaired MVK detoxification in *At*StR1- mutants. Support for this conclusion was obtained from experiments with *At*StR1- mutants overexpressing *35S:At*StR1, where *AtPR4* expression levels were again comparable with those of *Ath* Col-0 wt (Figure 8). Overexpressing *35S:At*StR2 in *At*StR1- mutants did not result in the attenuation of *AtPR4* expression. This showed that mainly *At*StR1 but not *At*StR2 is involved in MVK detoxification.

**Figure 9:**
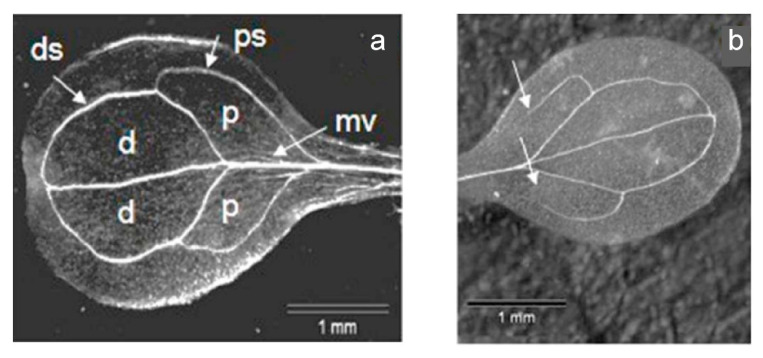
Vein patterning in cotyledons. (**a**) normal structure with distal (d) and proximal (p) areoles closed; (**b**) aberrant structure, with only the distal areoles closed. ps = proximal secondary vein; ds = distal secondary vein; mv = middle vein. *Ath* Col-0 wt (*n* = 217 seedlings) had 27.6% (8.8% pairs) and *At*StR1- mutant (*n* = 208 seedlings) had 42.3% (31.7% pairs) aberrant vein patterning, including the typical aberration shown in (**b**).

*At*StR1- mutant plants show a phenotype with altered vein patterning [5]. This phenotype could also be verified in the plants used in this study (Figure 9).

**Figure 10:**
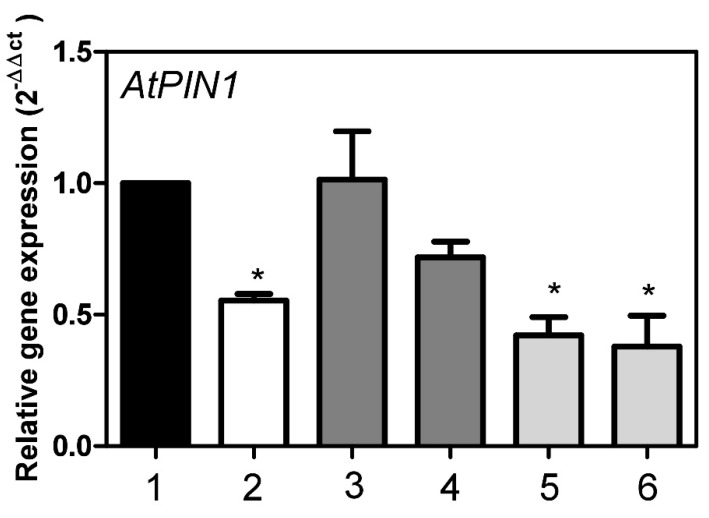
Expression of *PIN1* in *A. thaliana* Col-0 wt and mutant lines. (**1**) *Ath Col-0* wt, (**2**) *At*StR1- mutant, (**3**) *35S:At*StR1/*At*StR1-1, (**4**) *35S:At*StR1/*At*StR1-2, (**5**) *35S:At*StR2/*At*StR1-1, (**6**) *35S:At*StR2/*At*StR1-2**.** Mean ± STD (*n* = 3), normalized on *Act2* and *EF1α* (* = *p* < 0.05).

The expression of the auxin transporter *PIN1* is dependent on the availability of GSH, i.e., influenced by the tissue’s redox state. *PIN1*-deficient mutants also showed aberrant vein patterning [42]. We here demonstrated that *PIN1* expression was reduced by MVK to less than 30% of the control values after 3 h of treatment. *PIN1* expression was almost switched off after prolonged MVK treatment. In *At*StR1- mutant plants, *PIN1* expression levels were about 50% of the *Ath* Col-0 wt level. Curing the *At*StR1- mutant with *35S:At*StR1 yielded plants showing equally high *PIN1* expression as *Ath* Col-0 wt, whereas recovery was not observed in plants overexpressing *35S:At*StR2 (Figure 10).

All *Ath* Col-0 wt plants germinated in the presence of MVK (*n* = 60) displayed aberrations in vein patterning, whereas 50% of the cotyledons of non-treated controls looked like the one shown in Figure 9a. This might be in direct correlation with the reduced *PIN1* expression (about 70%) in MVK treated *Ath* Col-0 wt plants.

*At*StR1- mutant overexpressing *35S:At*StR1 or *35S:At*StR2 could not cure aberrant vein patterning (Figure S5). This may be due to the CaMV 35S promoter which constitutively expresses genes in all transformed tissues. The venation network in *Arabidopsis* leaves develops in a multistage process, which requires a sophisticated pattern of auxin transport through the leaf tissue. We demonstrated that the process is to some degree perturbed in the absence of functional *VEP1* and it seems likely that proper venation network development requires a proper spatiotemporal regulation of *VEP1* expression. This may explain why with restored global *PIN1* expression levels, constitutive overexpression of *VEP1* in our transgenic plants still does not sufficiently mimic the required pattern of VEP1 activity necessary to rescue the phenotype.

This implies that the role of *VEP1* in vein patterning is related to the ability of the encoded PRISE (*At*StR1) to reduce small reactive 1,4-enones, as demonstrated here for MVK released in stress reactions. Other PRISEs, like *At*StR2 in this study, may play completely different roles considering their individual expression patterns, their inability to cure symptoms caused by *At*StR1 deficiency and their different substrate specificities with a tendency to discriminate for larger molecules. RES are cytotoxic substances causing cell damage [20,38]. RES have also been demonstrated to activate defense-related gene expression and to be involved in signaling pathways ([21,43,44] among others). RES may therefore be regarded as archaic plant signals acting in a phytohormone-like manner. We here demonstrated that *At*StR1 gene knockout led to stronger responses of defense-related genes after exposure to MVK (Figure 8). We assume that PRISE-independent modulators of defense-related gene expression have evolved in plants.

In correlation with comparative metabolism and the evolution of specialized natural products, PRISEs encoded by *VEP1*-like genes may facilitate pathways leading to SNAPs [1] such as 5β-cardenolides [14] and iridoids [7]. Similarly, BAHD acyltransferases and aromatic prenyltransferases, involved in the biosynthesis of paclitaxel and monoterpenes, respectively, showed an expansion and high rates of evolution favoring some plant-specialized metabolic processes [45]. This might also be the case for the PRISE family in individual plant species.

Enzymes engaged in basic metabolism can be recruited into specialized metabolism in different plant lineages, their substrate promiscuity allowing these enzymes to contribute catalytic steps to new pathways if acceptable substrates are present. Promiscuous enzymes are important elements in pathway evolution and may complement other mechanisms such as changes in transcriptional regulation, defense strategies, changes in protein-protein interactions or protein folding [46].

## 3. Materials and Methods

### 3.1. Plant Material

An *At*StR1 T-DNA-insertion line (SALK 097798) was used to investigate the effects of *At*StR1 knockout. Plants derived from ordered seeds (Nottingham Arabidopsis Stock Centre; NASC) were tested for homozygous T-DNA insertion by PCR (T_A_ = 50 °C). Primers were derived by the online-tool “SALK T-DNA verification primer design” (http://signal.salk.edu/tdnaprimers.2.html (accessed on 17 April 2017); LBp1.3 ATT TTG CCG ATT TCG GAA C; LP097798 TTA TCG CCG TAA CCA CTT TTG; RP097798: AAA CAA CAA CGG AGA CCC TTC). Seeds from homozygous plants were stratified, germinated and cultivated as wild type (wt) plants in the greenhouse. Coding sequence of *At*StR1 (At2g24220) or *At*StR2 (At5g58750), respectively, was amplified using primers: JK_pEG^100^_StR1_for: TAT ACT CGA GAT GAA ACA TCA CCA TCA CCA TCA C; JK_pEG^100^_StR1_rev: AAT TTC TAG ATC AAG GTA CGA TCT TGA ACG C; JK_pEG^100^_StR2_for: TAT ACT CGA GAT GGG GTC TGA AAA TGG CAG; JK_pEG^100^_StR2_rev: AAT TTC TAG ATT ACA AAG GAA TGA GTT TTT CAT CT. *At*StR1 and *At*StR2 coding sequences were then cloned into pEarleyGate 100 (pEG^100^) using restriction enzymes XhoI and XbaI (New England Biolabs, Ipswich, MA, USA) and transformed into *E. coli* DH5α cells. Plasmids isolated from these cells were sequenced and those with verified constructs were used for the transformation of the *Agrobacterium tumefaciens* strain GV3101 (GV).

Transformed GVs carrying the cloned constructs were used for the transformation of SALK_097798 by floral-dip transformation [47]. Seeds of dipped plants were stratified and germinated as described above. One week after transfer into a climate chamber for germination, the seedlings were treated with glufosinate-ammonium (1 g L^−1^). This was repeated 3 times within 10 days. Surviving plants were tested for the integration of T-DNA by PCR with primers against bar gene (BAR for: TGC ACC ATC GTC AAC CAC TAC ATC GAG; BAR rev: CAG GCT GAA GTC CAG CTG). Plants with integrated T-DNA were transferred into the greenhouse for propagation. Seeds of these plants were stratified, germinated and cultivated as described above. All plants were tested for the integration of T-DNA before their use in experiments.

### 3.2. Heterologous Expression of AtStR2

In the restriction-free cloning of *At*StR2 (At5g58750) into expression vector pDEST17, the following primer pairs were used: forward primer: 5′TCGTACTACCATCACCATCACCATCACATGGGGTCTGAAAATGGCA′3. reverse primer: 5′GCCCCAAGGGGTTATGCTAGTTATTACAAAGGAATGAGTTTTTCATCT ′3.

Primer pairs were derived using rf-cloning.org [48]. The rf-cloning method [48] had successfully been used for integrating PRISEs into pDEST17 before by [14]. The restriction-free cloning strategy followed the procedures described previously [14,49] using *E. coli* DH5α cells for the primary transformation and *E. coli* soluBL21 for the recombinant protein expression. Correctly integrated coding sequence (in frame with His-Tag of pDEST17 vector) was confirmed by sequencing (Eurofins, Brussels, Belgium) using standard primers against T7 promoter and T7 terminator. *At*StR2 expression was induced in 1 L *E. coli* soluBL21 cultures (optical density of 0.5 to 0.7) by adding IPTG (final concentration of 0.1 mM). To avoid inclusion bodies [50] the culture was incubated at 4 °C for 96 h as described [13]. This was previously used for the expression of PRISEs [7]. Cells were harvested and protein was extracted as described before [14]. An ÄKTA purifier chromatography system (GE Healthcare, Upsala, Sweden) and HisTrap™ HP columns were used for the purification of recombinant *At*StR2 by immobilized metal-chelate chromatography, as described by [14]. SDS-PAGE and semidry immunoblotting (Figure S1) were carried out following QIAexpress Detection and Assay Handbook (QIAgen), which was used successfully for PRISEs by [7]. We used 12% Bis-Tris polyacrylamide gels for SDS-PAGEs and nitrocellulose membranes for electroblotting. Recombinant *At*StR2 was detected using mouse anti-His (mixture of RGS-, Tetra-, and Penta-His antibodies; dilution 1:2000; QiAgen, Hilden, Germany) and anti-mouse IgG-peroxidase antibodies (Sigma, Munich, Germany). As a result, we detected recombinant *At*StR2 as the chemiluminescence of 3-aminophtalate released from luminol.

### 3.3. Enzyme Kinetics

The reductase activity of recombinant *At*StR2 was measured spectrophotometrically by following the conversion of NADPH/H^+^ at 340 nm [7,14,32]. Enzyme kinetics were determined by using protein concentrations from 0.01 to 0.1 mg/mL of recombinant *At*StR2 (depending on the substrate analyzed), 0.2 mM NADPH/H^+^ and varying concentrations of the different substrates (0.05 to 1.6 mM) [14].

### 3.4. In Silico Analysis

The sequence alignment was generated using ClustalW [22]. The homology model of *At*StR2 was built on the crystal structure of *At*StR1 (Schmidt et al. 2018, PDB entry 6el3.1.A) with Swissmodel [29]. Docking simulations of progesterone were performed with AutoDock Vina [30]. The search area for appropriate docking was defined as a cube (side length = 30 Å), the coordinates (5/−5/−60) were optimized manually. UCSF Chimera [31] was used to visualize the docking calculations.

### 3.5. MVK Stress Treatment

A total of 16 µL of a MVK solution (Merck KGaA, Darmstadt, Germany) was diluted with 50 mL of water. Plants were transferred into a 1 L gas chamber. Diluted MVK was added to a total concentration of 2 µmol L^−1^ air volume and the chamber sealed during a 3 h incubation. Control plants were treated with tap water. Plant material was harvested after a 3 h treatment [14].

### 3.6. Cold Treatment

For cold treatment, plants were transferred into a climate chamber at 4 °C and treated for 36 h under long day conditions (16 h light/8 h dark). Control plants were cultivated in a climate chamber at 22 °C under long day conditions (16 h light/8 h dark). mRNA values of *At*StR1, *At*StR2 and *AtAct2* were shown on agarose gels of both control and stressed plants (Figure S3). Primers used in this experiment are presented in a data supplement (Supplementary Table S2).

### 3.7. RNA Isolation and cDNA-Synthesis

For RNA isolation, the 4 youngest leaves and the shoot apical meristem or 7 days old seedlings were used. Isolation was done with the use of Monarch^®^ Total RNA Miniprep Kit (New England Biolabs, Ipswich, MA, USA). gDNA was digested with a DNase I (New England Biolabs, Ipswich, USA) treatment. A total of 1500 µg of the resulting RNA was transcribed into cDNA by using a RevertAid H Minus First Strand cDNA Synthesis Kit (Thermo Fisher Scientific Inc., Waltham, MA, USA). cDNA was diluted 3:1 and used for quantitative real-time PCR.

### 3.8. Quantitative Real-Time PCR and Semiquantitative PCR

All quantitative real-time PCRs (qPCR) were realized in a Rotor-Gene Q thermocycler (Qiagen, Venlo, The Netherlands) using the following program (180 s at 95 °C followed by 40 cycles of 15 s at 95 °C; T_A_ 40 s) and Brilliant III Ultra-Fast SYBR^®^ Green QPCR Master Mix (Agilent Technologies, Santa Clara, CA, USA). Quantification of gene expression was calculated using the 2^−ΔΔct^-method [51]. Primers designed for quantitative real-time PCR are listed in a data supplement (Supplementary Table S1). Semiquantitative PCR was carried out in a peqSTAR 96 Universal Gradient thermocycler (Peqlab, Erlangen, Germany) using either primers shown in Supplementary Table S1 and a mRNA template isolated from adult leaves and seedlings, or primers shown in Supplementary Table S2 and an mRNA template extracted from adult leaves after cold treatment. The PCR program was designed as follows: 1 × 95 °C for 180 s, 40 cycles, 95 °C for 20 s, 50 °C for 30 s, 68 °C for 60 s, 1 × 68 °C for 600 s. *Taq* DNA Polymerase (New England Biolabs, Ipswich, MA, USA) was used for semiquantitative PCR. PCR products were separated on 1% agarose gels.

### 3.9. Quantification of Protein Activity in Plant Extracts

One gram of fresh leaves was snap frozen in liquid nitrogen. Frozen plant material was ground to a fine powder. To a total of 0.2 g of plant material the same amount of PVPP was added. Protein extraction from plant material followed the method of [52].

Total protein concentrations were determined according to [53]. Progesterone 5β-reductase activity was assayed in the presence of 1 mg mL^−1^ total leaf protein, 0.3 mM progesterone and a NADPH/H^+^-regenerating system (1.1 mM glucose 6-phosphate, 6.4 mM NADP+ and 4.2 nkat glucose-6-phosphate dehydrogenase) as described by [14,52]. To monitor the consumption of substrate we used a Shimadzu GC2010/QP-2010S in IE mode with helium as carrier gas (flow: 1.2 mL min^−1^) and DB-5ms columns (JW GC column) from Agilent (30 m × 0.25 mm × 0.25 µm). Progesterone and testosterone (internal standard) were from Steraloids Inc. (Newport, RI, USA). The program used is described by [14]. Additionally, products of the above-mentioned enzyme assays were blotted on TLC plates (silica gel 60; Merck) and run in GC–MS analysis (Figure S4), as described by [53].

### 3.10. Analysis of the Vein Patterning in Cotyledons

Plant tissue was fixed by immersion in a mixture of ethanol and acetic acid (3:1) overnight to remove chlorophyll and subsequently washed with water. The vein patterning was analyzed with an Axioskop HBO 50 (Zeiss).

### 3.11. Statistical Analysis

Experiments were conducted as three or more independent biological replicates with three technical replicates each. One-way analyses of variance (ANOVA followed by Tukey’s post hoc test) were used to compare the means of various groups. A post hoc Bonferroni correction was applied for multiple comparisons. *p* values ≤ 0.05 were considered as statistically significant and indicated by an asterisk. GraphPad Prism 8 Software (GraphPad) was used for data analysis and graph design.

## 4. Conclusions

Our results added evidence that *At*StR1, *At*StR2 and PRISEs of different clusters in general play different roles in stress and defense mechanisms. Based on our findings, we conclude that PRISEs are involved in the detoxification of small reactive 1,4-enones and are therefore part of the complex detoxification machinery of RES in plants. In the context of comparative metabolism and the evolution of specialized natural products (SNAPs), promiscuous enzymes, such as PRISEs, can provide important elements in pathway evolution. A comparison of closely related plant species, accumulating different sets of SNAPs but sharing homologues sets of genes/enzymes, adds a valuable new way of looking at comparative metabolism and “silent” or “underground” metabolism.

## Figures and Tables

**Table 1:** Enzyme activity of PRISEs from *A. thaliana*. The kinetic constants for the cosubstrate were determined using progesterone as the substrate.

Enzyme (Substrate)	K_m_ (µM)	*k_cat_* (s^−1^)	Catalytic Efficiency (s^−1^ M^−1^)
**r*At*StR2**			
r*At*StR2 (progesterone)	64.7	0.15	2314
r*At*StR2 (MVK)	157.5	0.03	163
r*At*StR2 (NADPH_2_)	41.0	0.12	2927
**Other PRISEs of cluster II**			
r*Ec*P5βR2 (progesterone) ^a^	82	0.05	552
r*Ec*P5βR2 (MVK) ^a^	224	0.05	245
r*Cr*P5βR6 (progesterone) ^a^	75.5	0.40	5530
r*Cr*P5βR6 (MVK) ^a^	118	0.03	261
**r*At*StR1 and r*At*St5βR1_F153A_F342A**			
r*At*StR1 (progesterone) ^b^	124.8	0.28	2244
r*At*StR1 (MVK) ^b^	75.5	0.20	7299
r*At*StR1_F153A_F342A (progesterone) ^c^	217	2.00	9218
r*At*StR1_F153A_F342A (MVK) ^c^	-	n.a.	-
**Other PRISEs of cluster I**			
r*Ec*P5βR1 (progesterone) ^b^	77	0.01	31
r*Ec*P5βR1 (MVK) ^b^	344	0.72	2143
r*Cr*P5βR4 (progesterone) ^b^	153	0.02	123
r*Cr*P5βR4 (MVK) ^b^	123	0.20	1562
**Other MVK-converting enzymes**			
*At*AER (MVK) ^d^	55.0	83.0	1,500,000
*At*AOR (MVK) ^e^	2880	74.0	25,700

^a^ [7]; ^b^ [9]; ^c^ [10]; ^d^ [26]; ^e^ [27]. n.a. = not accepted as substrate; *n* > 3.

## Data Availability

Data is contained within the article or supplementary material.

## Supplementary Materials

The following are available online at https://www.mdpi.com/article/10.3390/metabo12010011/s1, Figure S1: SDS-PAGE and Western blot of r*At*StR2, Figure S2: Enantioselective conversion of progesterone by r*At*StR2, Figure S3: Expression of *At*StR1 and *At*StR2 after cold shock treatment, Figure S4: Analysis of progesterone 5β-reductase activity assayed in plant leaf extracts of *Ath* Col-0 wt and mutant lines, Figure S5: Analysis of vein patterning in *Ath* Col-0 wt and mutant lines, Table S1: List of primer pairs for quantitative real-time PCR experiments, Table S2: List of primers used in cold treatment experiment.

## References

[1] Kreis W., Munkert J. (2019). Exploiting enzyme promiscuity to shape plant specialized metabolism. J. Exp. Bot..

[2] Noda-Garcia L., Liebermeister W., Tawfik D.S. (2018). Metabolite-Enzyme Coevolution: From Single Enzymes to Metabolic Pathways and Networks. Annu. Rev. Biochem..

[3] Djoumbou-Feunang Y., Fiamoncini J., Gil-de-la-Fuente A., Greiner R., Manach C., Wishart D.S. (2019). BioTransformer: A comprehensive computational tool for small molecule metabolism prediction and metabolite identification. J. Cheminform..

[4] (2019). EFSA Workshop on in vitro comparative metabolism studies in regulatory pesticide risk assessment. EFSA Support. Publ..

[5] Jun J.H., Ha C.M., Nam H.G. (2002). Involvement of the *VEP1* gene in vascular strand development in *Arabidopsis thaliana*. Plant Cell Physiol..

[6] Yang K.Y., Moon Y.H., Choi K.H., Kim Y.H., Eun M.Y., Guh J.O., Kim K.C., Cho B.H. (1997). Structure and expression of the AWI 31 gene specifically induced by wounding in *Arabidopsis thaliana*. Mol. Cells.

[7] Munkert J., Pollier J., Miettinen K., van Moerkercke A., Payne R., Müller-Uri F., Burlat V., O’Connor S.E., Memelink J., Kreis W. (2015). Iridoid synthase activity is common among the plant progesterone 5β-reductase family. Mol. Plant.

[8] Herl V., Fischer G., Reva V.A., Stiebritz M., Muller Y.A., Müller-Uri F., Kreis W. (2009). The VEP1 gene (At4g24220) encodes a short-chain dehydrogenase/reductase with 3-oxo-Delta4,5-steroid 5beta-reductase activity in *Arabidopsis thaliana* L.. Biochimie.

[9] Schmidt K., Petersen J., Munkert J., Egerer-Sieber C., Hornig M., Muller Y.A., Kreis W. (2018). PRISEs (progesterone 5β-reductase and/or iridoid synthase-like 1,4-enone reductases): Catalytic and substrate promiscuity allows for realization of multiple pathways in plant metabolism. Phytochemistry.

[10] Petersen J., Lanig H., Munkert J., Bauer P., Müller-Uri F., Kreis W. (2016). Progesterone 5β-reductases/iridoid synthases (PRISE): Gatekeeper role of highly conserved phenylalanines in substrate preference and trapping is supported by molecular dynamics simulations. J. Biomol. Struct. Dyn..

[11] Gärtner D.E., Keilholz W., Seitz H.U. (1994). Purification, characterization and partial peptide microsequencing of progesterone 5 beta-reductase from shoot cultures of *Digitalis purpurea*. Eur. J. Biochem..

[12] Roca-Pérez L., Boluda R., Gavidia I., Pérez-Bermúdez P. (2004). Seasonal cardenolide production and *Dop5βr* gene expression in natural populations of *Digitalis obscura*. Phytochemistry.

[13] Herl V., Fischer G., Mueller-Uri F., Kreis W. (2006). Molecular cloning and heterologous expression of progesterone 5β-reductase from *Digitalis lanata* Ehrh. Phytochemistry.

[14] Klein J., Horn E., Ernst M., Leykauf T., Leupold T., Dorfner M., Wolf L., Ignatova A., Kreis W., Munkert J. (2021). RNAi-mediated gene knockdown of progesterone 5β-reductases in *Digitalis lanata* reduces 5β-cardenolide content. Plant Cell Rep..

[15] Geu-Flores F., Sherden N.H., Courdavault V., Burlat V., Glenn W.S., Wu C., Nims E., Cui Y., O’Connor S.E. (2012). An alternative route to cyclic terpenes by reductive cyclization in iridoid biosynthesis. Nature.

[16] Nguyen T.-D., O’Connor S.E. (2020). The Progesterone 5β-Reductase/Iridoid Synthase Family: A Catalytic Reservoir for Specialized Metabolism across Land Plants. ACS Chem. Biol..

[17] Tarrío R., Ayala F.J., Rodríguez-Trelles F. (2011). The Vein Patterning 1 (VEP1) gene family laterally spread through an ecological network. PLoS ONE.

[18] Burda E., Kraußer M., Fischer G., Hummel W., Müller-Uri F., Kreis W., Gröger H. (2009). Recombinant Δ4,5-Steroid 5 β-Reductases as Biocatalysts for the Reduction of Activated C=C-Double Bonds in Monocyclic and Acyclic Molecules. Adv. Synth. Catal..

[19] Durchschein K., Wallner S., Macheroux P., Schwab W., Winkler T., Kreis W., Faber K. (2012). Nicotinamide-Dependent Ene Reductases as Alternative Biocatalysts for the Reduction of Activated Alkenes. Eur. J. Org. Chem..

[20] Kai H., Hirashima K., Matsuda O., Ikegami H., Winkelmann T., Nakahara T., Iba K. (2012). Thermotolerant cyclamen with reduced acrolein and methyl vinyl ketone. J. Exp. Bot..

[21] Alméras E., Stolz S., Vollenweider S., Reymond P., Mène-Saffrané L., Farmer E.E. (2003). Reactive electrophile species activate defense gene expression in *Arabidopsis*. Plant J..

[22] Madeira F., Park Y.M., Lee J., Buso N., Gur T., Madhusoodanan N., Basutkar P., Tivey A.R.N., Potter S.C., Finn R.D. (2019). The EMBL-EBI search and sequence analysis tools APIs in 2019. Nucleic Acids Res..

[23] Thorn A., Egerer-Sieber C., Jäger C.M., Herl V., Müller-Uri F., Kreis W., Muller Y.A. (2008). The crystal structure of progesterone 5β-reductase from *Digitalis lanata* defines a novel class of short chain dehydrogenases/reductases. J. Biol. Chem..

[24] Pérez-Bermúdez P., García A.A.M., Tuñón I., Gavidia I. (2010). *Digitalis purpurea* P5βR2, encoding steroid 5β-reductase, is a novel defense-related gene involved in cardenolide biosynthesis. New Phytol..

[25] Rieck C., Geiger D., Munkert J., Messerschmidt K., Petersen J., Strasser J., Meitinger N., Kreis W. (2019). Biosynthetic approach to combine the first steps of cardenolide formation in *Saccharomyces cerevisiae*. Microbiologyopen.

[26] Mano J., Torii Y., Hayashi S., Takimoto K., Matsui K., Nakamura K., Inzé D., Babiychuk E., Kushnir S., Asada K. (2002). The NADPH:quinone oxidoreductase P1-ζ-crystallin in *Arabidopsis* catalyzes the α,β-hydrogenation of 2-alkenals: Detoxication of the lipid peroxide-derived reactive aldehydes. Plant Cell Physiol..

[27] Yamauchi Y., Hasegawa A., Taninaka A., Mizutani M., Sugimoto Y. (2011). NADPH-dependent reductases involved in the detoxification of reactive carbonyls in plants. J. Biol. Chem..

[28] Berman H.M., Westbrook J., Feng Z., Gilliland G., Bhat T.N., Weissig H., Shindyalov I.N., Bourne P.E. (2000). The Protein Data Bank. Nucleic Acids Res..

[29] Waterhouse A., Bertoni M., Bienert S., Studer G., Tauriello G., Gumienny R., Heer F.T., de Beer T.A.P., Rempfer C., Bordoli L. (2018). SWISS-MODEL: Homology modelling of protein structures and complexes. Nucleic Acids Res..

[30] Trott O., Olson A.J. (2010). AutoDock Vina: Improving the speed and accuracy of docking with a new scoring function, efficient optimization, and multithreading. J. Comput. Chem..

[31] Pettersen E.F., Goddard T.D., Huang C.C., Couch G.S., Greenblatt D.M., Meng E.C., Ferrin T.E. (2004). UCSF Chimera—A visualization system for exploratory research and analysis. J. Comput. Chem..

[32] Bauer P., Munkert J., Brydziun M., Burda E., Müller-Uri F., Gröger H., Muller Y.A., Kreis W. (2010). Highly conserved progesterone 5β-reductase genes (P5βR) from 5 beta-cardenolide-free and 5β-cardenolide-producing angiosperms. Phytochemistry.

[33] Tropper M., Höhn S., Wolf L.-S., Fritsch J., Kastner-Detter N., Rieck C., Munkert J., Meitinger N., Lanig H., Kreis W. (2021). 21-Hydroxypregnane 21-O-malonylation, a crucial step in cardenolide biosynthesis, can be achieved by substrate-promiscuous BAHD-type phenolic glucoside malonyltransferases from *Arabidopsis thaliana* and homolog proteins from *Digitalis lanata*. Phytochemistry.

[34] Yin L., Mano J., Tanaka K., Wang S., Zhang M., Deng X., Zhang S. (2017). High level of reduced glutathione contributes to detoxification of lipid peroxide-derived reactive carbonyl species in transgenic Arabidopsis overexpressing glutathione reductase under aluminum stress. Physiol. Plant..

[35] Lewinsohn E., Gijzen M. (2009). Phytochemical diversity: The sounds of silent metabolism. Plant Sci..

[36] Jardine K.J., Meyers K., Abrell L., Alves E.G., Serrano A.M.Y., Kesselmeier J., Karl T., Guenther A., Chambers J.Q., Vickers C. (2013). Emissions of putative isoprene oxidation products from mango branches under abiotic stress. J. Exp. Bot..

[37] Cappellin L., Loreto F., Biasioli F., Pastore P., McKinney K. (2019). A mechanism for biogenic production and emission of MEK from MVK decoupled from isoprene biosynthesis. Atmos. Chem. Phys..

[38] Vollenweider S., Weber H., Stolz S., Chételat A., Farmer E.E. (2000). Fatty acid ketodienes and fatty acid ketotrienes: Michael addition acceptors that accumulate in wounded and diseased Arabidopsis leaves. Plant J..

[39] Horiyama S., Hatai M., Ichikawa A., Yoshikawa N., Nakamura K., Kunitomo M. (2018). Detoxification Mechanism of α,β-Unsaturated Carbonyl Compounds in Cigarette Smoke Observed in Sheep Erythrocytes. Chem. Pharm. Bull..

[40] Ernst M. (2015). Untersuchungen zur physiologischen Funktion der Progesteron-5β-Reduktase in Arabidopsis thaliana und Vitis vinifera. Ph.D. Thesis.

[41] Harvey C.M., Sharkey T.D. (2016). Exogenous isoprene modulates gene expression in unstressed *Arabidopsis thaliana* plants. Plant Cell Environ..

[42] Bashandy T., Guilleminot J., Vernoux T., Caparros-Ruiz D., Ljung K., Meyer Y., Reichheld J.-P. (2010). Interplay between the NADP-linked thioredoxin and glutathione systems in Arabidopsis auxin signaling. Plant Cell.

[43] Biswas M.S., Fukaki H., Mori I.C., Nakahara K., Mano J. (2019). Reactive oxygen species and reactive carbonyl species constitute a feed-forward loop in auxin signaling for lateral root formation. Plant J..

[44] Mano J., Biswas M.S., Sugimoto K. (2019). Reactive Carbonyl Species: A Missing Link in ROS Signaling. Plants.

[45] Kusano H., Li H., Minami H., Kato Y., Tabata H., Yazaki K. (2019). Evolutionary Developments in Plant Specialized Metabolism, Exemplified by Two Transferase Families. Front. Plant Sci..

[46] Moghe G.D., Last R.L. (2015). Something Old, Something New: Conserved Enzymes and the Evolution of Novelty in Plant Specialized Metabolism. Plant Physiol..

[47] Clough S.J., Bent A.F. (1998). Floral dip: A simplified method for *Agrobacterium*-mediated transformation of *Arabidopsis thaliana*. Plant J..

[48] Bond S.R., Naus C.C. (2012). RF-Cloning.org: An online tool for the design of restriction-free cloning projects. Nucleic Acids Res..

[49] Unger T., Jacobovitch Y., Dantes A., Bernheim R., Peleg Y. (2010). Applications of the Restriction Free (RF) cloning procedure for molecular manipulations and protein expression. J. Struct. Biol..

[50] Stevens R.C. (2000). Design of high-throughput methods of protein production for structural biology. Structure.

[51] Livak K.J., Schmittgen T.D. (2001). Analysis of relative gene expression data using real-time quantitative PCR and the 2(−ΔΔC(T)) Method. Methods.

[52] Ernst M., de Padua R.M., Herl V., Müller-Uri F., Kreis W. (2010). Expression of 3β-HSD and P5βR, genes respectively coding for Δ5-3β-hydroxysteroid dehydrogenase and progesterone 5β-reductase, in leaves and cell cultures of *Digitalis lanata* EHRH. Planta Med..

[53] Bradford M.M. (1976). A rapid and sensitive method for the quantitation of microgram quantities of protein utilizing the principle of protein-dye binding. Anal. Biochem..

