# Supplementary material for: Knockout of Arabidopsis thaliana VEP1, Encoding a PRISE (Progesterone 5β-Reductase/Iridoid Synthase-Like Enzyme), Leads to Metabolic Changes in Response to Exogenous Methyl Vinyl Ketone (MVK)
Source: Metabolites. 2021 Dec 23;12(1):11. doi: 10.3390/metabo12010011 (PMC8778713; doi:10.3390/metabo12010011)
Supplement: Supplementary file 1 [file metabolites-12-00011-s001.zip › metabolites-1421595-supplementary.pdf]

metabolites

Knockout of *Arabidopsis thaliana VEP1*, Encoding a PRISE (Progesterone 5β-Reductase/Iridoid Synthase-Like Enzyme), Leads to Metabolic Changes in Response to Exogenous Methyl Vinyl Ketone (MVK)

Jan Klein [1], Mona Ernst [2], Alexander Christmann [3], Marina Tropper [2], Tim Leykauf [2], Wolfgang Kreis [2] and Jennifer Munkert [2,*]

[1] Department of Plant Physiology, Matthias-Schleiden-Institute for Genetics, Bioinformatics and Molecular Botany, University of Jena, 07743 Jena, Germany; jan.klein@uni-jena.de
[2] Department of Biology, University of Erlangen-Nuremberg, 91058 Erlangen, Germany; mona.ernst@gmx.de (M.E.); marina.tropper@fau.de (M.T.); tim.leykauf@fau.de (T.L.); wolfgang.kreis@fau.de (W.K.)
[3] Chair of Botany, TUM School of Life Sciences Weihenstephan, Technical University of Munich, 85354 Freising, Germany; alexander.christmann@tum.de
* Correspondence: jennifer.munkert@fau.de; Tel.: +49-91318528251

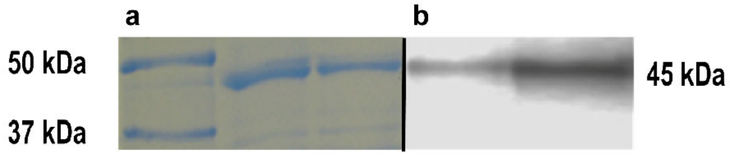

Figure S1. Heterologous expression of CDS of *AtStR*2 in pDEST17 vector in *E. coli*. (a) SDS-Page analysis of recombinant *At*StR2. Purified r*At*StR2 protein has a size of about 43 kDa and was visualized with Coomassie-Brilliant-Blue R 250; (b) Immunoblot analysis of r*At*StR2 using anti-His antibodies (primary) and anti-mouse IgG-peroxidase antibodies (secondary). Chemiluminescence was used for detection.

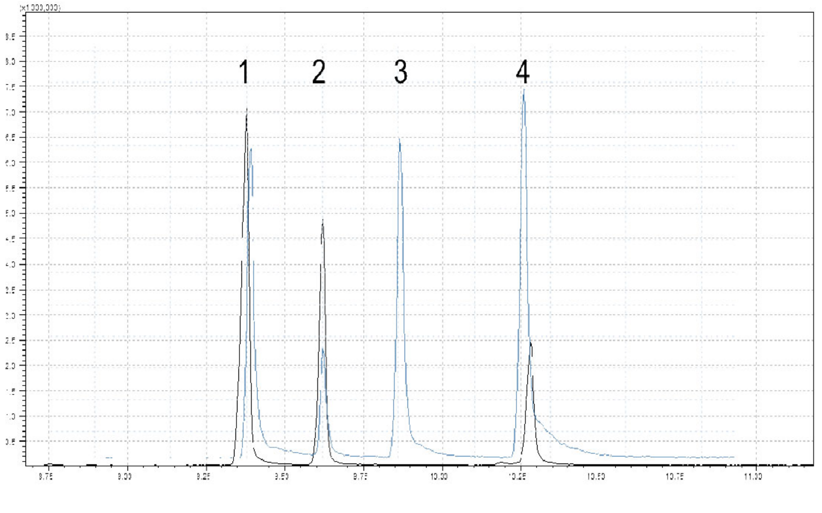

Figure S2. Enantioselective conversion of progesterone by r*At*St5βR2. Steroid mixture (blue) including testosterone (1; t_{Rt} = 9.3 min), 5β-pregnane-3,20-dione (2; t_{Rt} = 9.6 min), 5α-pregnane-3,20-dione (3; t_{Rt} = 9.9 min) and progesterone (4; t_{Rt} = 10.3 min) by the GC-MS method used to determine product specificity of r*At*StR2 (black).

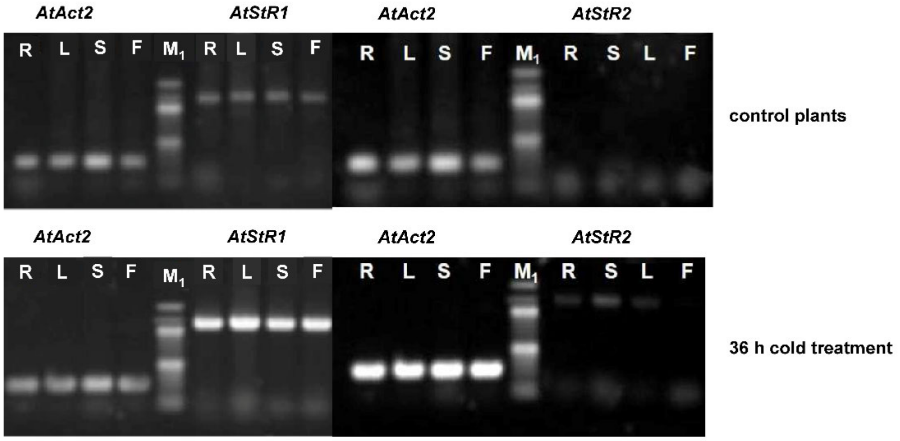

Figure S3. Expression of *AtStR1* and *AtStR2* in normal condition and after cold shock treatment. M1 = 100 bp marker; Expression of *AtAct2*, *AtStR1* and *AtStR2* in roots (R), leaves (L), stem (S) and flowers (F) of *Arabidopsis thaliana* in control plants and after 36 h cold treatment.

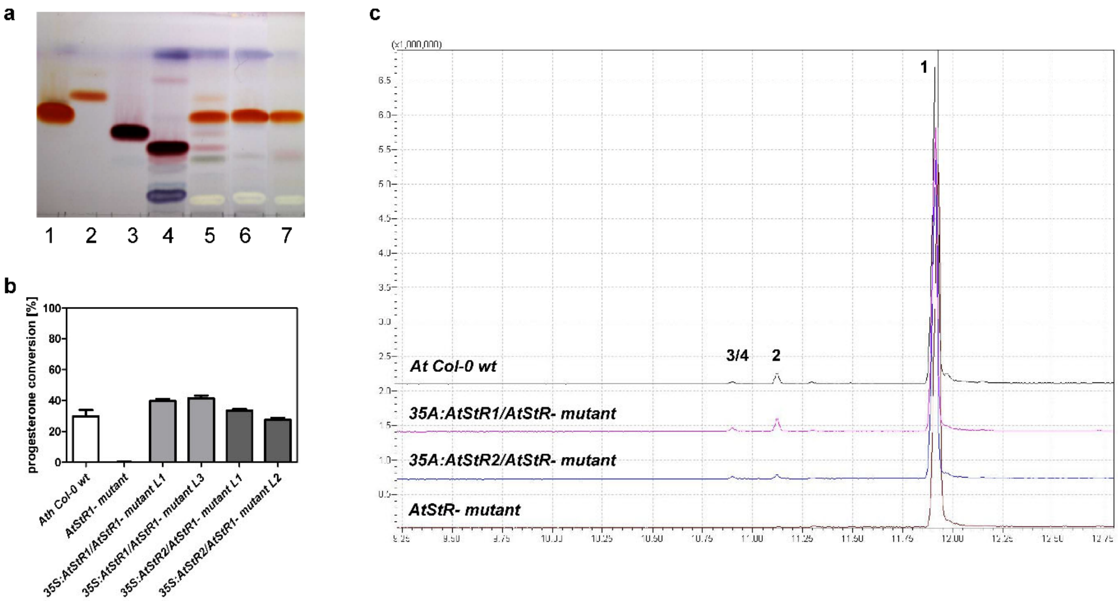

Figure S4. *At*StR protein activity in plant leaf extracts of *Ath* Col-0 wt plants (5), *AtStR-* (6), *35S:AtStR1/AtStR1−* and in *35S:AtStR2/AtStR1−* mutant lines. (a) Thin-layer chromatography analysis of standard compounds: (1) progesterone (Rf = 0.5), (2) 5β-pregnane-3,20-dione (Rf = 0.6), (3) 5β-pregnan-3β-ol-20-one (Rf = 0.4), (4) 5β-pregnan-3α-ol-20-one (Rf = 0.3), *At*StR protein activity in plant leaf extracts of *Ath* Col-0 wt plants (5) and (6) *AtStR−* mutant, (8) heat inactivated control assay; (b) quantification of converted progesterone [%] in wt and mutant lines; (c) GC-MS analysis of protein assays from wt and mutant lines. (1) progesterone, (2) 5β-pregnane-3,20-dione, (3) 5β-pregnan-3β-ol-20-one, (4) 5β-pregnan-3α-ol-20-one.

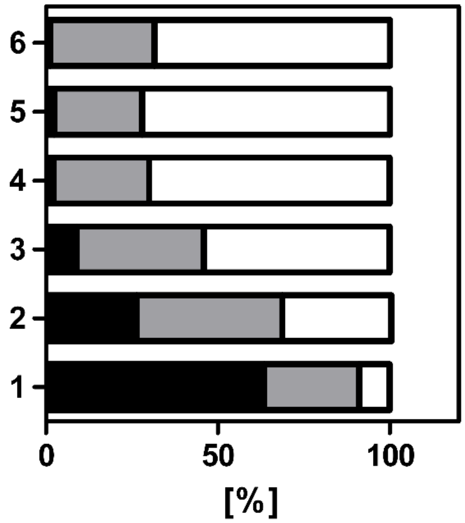

Figure S5. Vein patterning in cotyledons in *Ath* Col-0 wt plants, *AtStR-*, *35S:AtStR1/AtStR1-* and in *35S:AtStR2/AtStR1-* mutant lines. Vein patterning is analyzed in % regarding intact and normal structure with distal and proximal areoles closed (black), aberrant vein pattering structure (grey) or aberrant structure in pairs (white). (1) *Ath* Col-0 wt, (2) *AtStR1−*, (3) *35S:AtStR1/AtStR1-1*, (4) *35S:AtStR1/AtStR1*-2, (5) *35S:AtStR2/AtStR1-1*, (6) *35S:AtStR2/AtStR1-2*.

Table S1. List of primer used in qPCR.

Name	Oligonucleotide	Annealing Temperature	Locus
JK_qAtAct_for:	5′TCAGATGCCCAGAAGTCTTGTT′3	57°C	At3g18780
JK_qAtAct_rev:	5′GAGATCCACATCTGCTGGAATG′3		
JK_qAtEF1α_for:	5′GTACGTCGCATCCAACTCCA′3	57°C	At1g18070
JK_qAtEF1α_rev:	5′CACAGCGAAACGTCCAAGTG′3		
JK_qAtStR1_for:	5′ CTCCCTCTTTCCGACACACC ′3	60° C	At4g24220
JK_qAtStR1_rev:	5′ GTCAACGTTGGTGAAAGGGC ′3		
JK_qAtStR2_for:	5′CAATGGCTCGTGGAGAAGGT′3	60° C	At5g58750
JK_qAtStR2_rev:	5′TTCCGGCCAAATCTCCTTCC′3		
JK_qAtPR4_for:	5′TCAGATGCCCAGAAGTCTTGTT′3	57°C	At3g04720
JK_qAtPR4_rev:	5′GAGATCCACATCTGCTGGAATG′3		
JK_qAtGR1_for:	5′CGTGGAATGGGTGCTACTGT′3	60° C	At3g24170
JK_qAtGR1_rev:	5′TCGTCAACCTTCACAGCTCC′3		
JK_AtPIN1_for:	5′AGGGATGTTTTCGCCCAACA′3	57° C	At1g73590
JK_AtPIN1_rev:	5′ACCGTCCGTTGCCAATACTT′3		
JK_AtAER_for:	5′CACCACCGTCGAACTTAGGG′3	60° C	At5g16970
JK_AtAER_rev:	5′ATGCGCGTGAGTCATTGGA′3		
JK_qAtAOR_for	5′AACAACCACCGCCACAACT′3	57 °C	At1g23740
JK_qAtAOR_rev	5′TCAGAACATCAACTCCGCCG′3		

Table S2. List of primer used in cold treatment experiment.

Name	Oligonucleotide	Product Length	Locus
ME_qAtAct_for:	5´GGAGATCCACATCTGCTGGAATGT´3	312 bp	At3g18780
ME_qAtAct_rev:	5´ATTCAGGATGCCCAGAAGTCTTGTT´3		
ME_AtStR1_for:	5´CACCATGAGTTGGTGGTGGCTG´3	1167 bp	At4g24220
ME_AtStR1_rev:	5´ TCAAGGTACGATCTTGAACGCCTT´3		
ME_AtStR2_for:	5´ATGGGGTCTGAAAATGGCAG´3	1161 bp	At5g58750
ME_AtStR2_rev:	5´TTACAAAGGAATGAGTTTTTCATCT´3		
